# Supplementary material for: Prevalence of migraine in adults with celiac disease: A case control cross-sectional study
Source: PLoS One. 2021 Nov 17;16(11):e0259502. doi: 10.1371/journal.pone.0259502 (PMC8598245; doi:10.1371/journal.pone.0259502)
Supplement: S1 Checklist — (PDF) [file pone.0259502.s001.pdf]

S3 Checklist. Checklist for data collection

Name:

Age:

Gender:

Drug history:

Has a history of head trauma or stroke or brain tumor?

Yes

No

1- Have you had a history of recurrent headaches throughout your life?

Yes

No (if the answer is yes answer the following questions)

2- How many times a week do you have headaches?

3- How long does each headache attack last?

Less than 4 hours

4-72 hours

More than 72 hours

4- Does your headache interfere with your daily activities?

Yes

No

5- How do you assess the severity of your headache?

Mild

Moderate

Severe

6- Do you have nausea and vomiting during a headache?

Yes

No

7- Does light bother you and aggravate your headache?

Yes

No

8- Does noise bother you and aggravate your headache?

Yes

No

9- Is your headache…

Unilateral

Bilateral

10- Is your headache…

Pulsatile

Non-pulsatile

11- Do you have any visual symptoms during the headache attack, such as seeing light or black spots, etc.?

Yes

No

12- Regarding the question number 11, how long do these symptoms last?

Less than 5 minutes

5-60 minutes

More than 60 minutes

13- Regarding the question number 11, is the symptom…

Unilateral

Bilateral

14- Regarding the question number 11, these symptoms occur in how many headache attacks?

One

More than one

15- Have you ever had sensory disturbances such as tingling or numbness during a headache?

Yes

No

16- Regarding the question number 15, how long do these symptoms last?

Less than 5 minutes

5-60 minutes

More than 60 minutes

17- Regarding the question number 15, is the symptom…

Unilateral

Bilateral

18- Regarding the question number 11, these symptoms occur in how many headache attacks?

One

More than one

19- Have you ever had a speech disorder during a headache?

Yes

No

20- Regarding the question number 19, how long do these symptoms last?

Less than 5 minutes

5-60 minutes

More than 60 minutes

21- Regarding the question number 19, these symptoms occur in how many headache attacks?

One

More than one
