# Supplementary material for: Prevalence of migraine in adults with celiac disease: A case control cross-sectional study
Source: PLoS One. 2021 Nov 17;16(11):e0259502. doi: 10.1371/journal.pone.0259502 (PMC8598245; doi:10.1371/journal.pone.0259502)
Supplement: S1 Text — (PDF) [file pone.0259502.s002.pdf]

Supporting information

S1 Text. Diagnostic criteria for migraine without aura

Diagnostic criteria:

A. At least five attacks1 fulfilling criteria B–D

B. Headache attacks lasting 4-72 hours (untreated or unsuccessfully treated)

C. Headache has at least two of the following four characteristics:

1. Unilateral location

2. Pulsating quality

3. Moderate or severe pain intensity

4. Aggravation by or causing avoidance of routine physical activity (e.g. walking or climbing stairs)

D. During headache at least one of the following:

1. Nausea and/or vomiting

2. Photophobia and phonophobia

E. Not better accounted for by another ICHD-3 diagnosis.
