# Supplementary material for: Prevalence of migraine in adults with celiac disease: A case control cross-sectional study
Source: PLoS One. 2021 Nov 17;16(11):e0259502. doi: 10.1371/journal.pone.0259502 (PMC8598245; doi:10.1371/journal.pone.0259502)
Supplement: S2 Text — (PDF) [file pone.0259502.s003.pdf]

S2 Text. Diagnostic criteria for migraine with typical aura

Description:

Migraine with aura in which aura consists of visual and/or sensory and/or speech/language symptoms, but no motor weakness, and is characterized by gradual development, duration of each symptom no longer than 1 hour, a mix of positive and negative features and complete reversibility.

Diagnostic criteria:

A. At least two attacks fulfilling criteria B and C

B. Aura consisting of visual, sensory and/or speech/ language symptoms, each fully reversible, but no motor, brainstem or retinal symptoms

C. At least two of the following four characteristics:

1. At least one aura symptom spreads gradually over ≥5 minutes, and/or two or more symptoms occur in succession

2. Each individual aura symptom lasts 5-60 minutes

3. At least one aura symptom is unilateral

4. The aura is accompanied, or followed within 60 minutes, by headache

D. Not better accounted for by another ICHD-3 diagnosis

Reference:

Cephalalgia 33(9) 629–808 International Headache Society 2013 Reprints and permissions:

sagepub.co.uk/journalsPermissions.nav; DOI: 10.1177/0333102413485658; cep.sagepub.com
