# Supplementary material for: Prevalence of migraine in adults with celiac disease: A case control cross-sectional study
Source: PLoS One. 2021 Nov 17;16(11):e0259502. doi: 10.1371/journal.pone.0259502 (PMC8598245; doi:10.1371/journal.pone.0259502)
Supplement: Attachment — Submitted filename: renamed_fa79c.docx [file pone.0259502.s004.docx]

MY COMMENTS:

Comment 1: The sex distribution were differed ( p= 0.000) between celiac and control groups (Table 1), this should be one of the limitations of this study.

Comment 2: Line 117 " Patients who reported a history of headaches..."; Line 132 " reported chronic headache..." . These two descriptions were inconsistent and should be clarified. "Headache " should be clearly defined, do you mean primary headache only, or secondary headache also included? "Chronic headache" is an ambiguous term, I am not able to know the meaning ( chronic primary headache history? or chronic daily headache? or others?) .

Comment 3: The authors didn't let us known how to perform the ICHD-3 based checklist (line 118) during questioning the patients, is it single-blinded or double-blinded?

Comment 4: According to Table 3, the both groups with more bilateral characters ( 67.2% and 65.2%) , this finding is different from the characters of general migraine patients, which "unilateral" were much more than "bilateral". This point should be clearly explained.

Comment 5: Table 5 revealed the two groups were differed in type I DM, abdominal pain, constipation and diarrhea, the possible causes of these differences needs to be explained.

Comment 6:Table 5 only compared CD subjects with migraine and non-migraine headache, however, it would be more interesting to compare the "symptoms and associated conditions" between CD with migraine headache and controls with migraine headache. If the authors have the data, please analyze and show in results and discussing them.

Comment 7: Based on comment 6, and the other results in this study, it's better to address the different characters between CD with migraine and migraine without CD (controls with migraine), and then provide possible explanations for them, in this way, this article may be more valuable.
