# Supplementary material for: Prevalence of migraine in adults with celiac disease: A case control cross-sectional study
Source: PLoS One. 2021 Nov 17;16(11):e0259502. doi: 10.1371/journal.pone.0259502 (PMC8598245; doi:10.1371/journal.pone.0259502)
Supplement: Attachment — Submitted filename: Response to reviewer.docx [file pone.0259502.s005.docx]

Responses to the reviewers:

***Reviewr1:***

Q1: Dose authors happen to have any data of ethnic background of this study?

A1: No

Q2: Q2
I could not find this paper at Reference of this paper.
Could you please consider adding this paper to Reference of this study?
Headache. 2013 Feb;53(2):344-55.
Prevalence of migraine in patients with celiac disease and inflammatory bowel disease
Alexandra K Dimitrova 1, Ryan C Ungaro, Benjamin Lebwohl, Suzanne K Lewis, Christina A Tennyson, Mark W Green, Mark W Babyatsky, Peter H Green

A2: This reference No. is 22.

***Reviewer*** ***2***:

All items mentioned by the 2^nd^ reviewer were considered.

***Reviewer*** ***3***:

Most items mentioned by the 3^rd^ reviewer were considered.

Table 4 was corrected; figures and percent are related to unilateral headaches.

For some unknown reasons the prevalence of some diseases and symptoms are different in CD with and without migraine.
