# Supplementary material for: Prevalence of migraine in adults with celiac disease: A case control cross-sectional study
Source: PLoS One. 2021 Nov 17;16(11):e0259502. doi: 10.1371/journal.pone.0259502 (PMC8598245; doi:10.1371/journal.pone.0259502)
Supplement: Attachment — Submitted filename: Response to Reviewers.docx [file pone.0259502.s006.docx]

Responses to the Reviewers:

***Reviewr1:***

Q1: Dose authors happen to have any data of ethnic background of this study?

A1: No

Q2: I could not find this paper at Reference of this paper.
Could you please consider adding this paper to Reference of this study?
Headache. 2013 Feb;53(2):344-55.
Prevalence of migraine in patients with celiac disease and inflammatory bowel disease
Alexandra K Dimitrova 1, Ryan C Ungaro, Benjamin Lebwohl, Suzanne K Lewis, Christina A Tennyson, Mark W Green, Mark W Babyatsky, Peter H Green

A2: This reference No. is 22.

***Reviewer*** ***2***:

As you mentioned, the terms “migraine headache” and “non-migraine headache” were replaced by the “migraine” and “non- migrainous headache” throughout the manuscript.

Conclusion was revised in “abstract” and “conclusion” sections (Lines 58-60 and Lines 268-271)

***Reviewer*** ***3***:

Comment 1: As you mentioned, the sex distribution was differed (P= 0.000) between celiac and control groups, and that was one of the limitations of this study and we mentioned that in discussion (line 258)

Comment 2: As you said, the term “chronic headache” was replaced. We mentioned in the method section. (Line 122-125)

Comment 3: The ICHD-3 based checklist was written in Supporting information caption and it was filled single-blinded.

Comment 4: Table 4 was revised; figures and percent are related to unilateral headaches.

Comment 5: GI symptoms and migraine were explained (lines 238-243, discussion section). Migraine and type 1 were explained. (Lines 86-90, Introduction section/ lines 244-247, discussion section)

Comment 6 & 7: Thanks for your good comment. Unfortunately, this data is not available in the control group.
