# Supplementary material for: Prevalence of migraine in adults with celiac disease: A case control cross-sectional study
Source: PLoS One. 2021 Nov 17;16(11):e0259502. doi: 10.1371/journal.pone.0259502 (PMC8598245; doi:10.1371/journal.pone.0259502)
Supplement: Attachment — Submitted filename: Responses to the Reviewers.docx [file pone.0259502.s007.docx]

Responses to the Reviewers:

***Reviewer*** ***2***:

As you mentioned, the term “primary headache” was replaced by the “headache” in manuscript.

According to ICHD-3, abdominal pain can be one of the episodic syndromes associated with migraine. (The International Classification of Headache Disorders, 3rd edition page 653)

***Reviewer*** ***3***:

In data collection, nausea and vomiting were both considered as a single manifestation and were not considered individually.

In data collection, photophobia and phonophobia were both considered as a single manifestation and were not considered individually.
