# Supplementary material for: Prevalence of migraine in adults with celiac disease: A case control cross-sectional study
Source: PLoS One. 2021 Nov 17;16(11):e0259502. doi: 10.1371/journal.pone.0259502 (PMC8598245; doi:10.1371/journal.pone.0259502)
Supplement: Attachment — Submitted filename: Responses to the Reviewers.docx [file pone.0259502.s008.docx]

Responses to the Reviewers:

***Reviewer*** ***3***:

Thanks for your comment, as you mentioned, based on the ICHD 3, (the diagnostic criteria of migraine without aura, the criteria D) in data collection, photophobia and phonophobia were both considered as a single manifestation and were not considered individually.

In Table 4, the term "photophobia and phonophobia" is correct and the word "or" was incorrectly mentioned, which was corrected.
